# Characterisation and molecular detection of *Echinococcus granulosus* in Duhok Governorate

**DOI:** 10.2478/helm-2026-0005

**Published:** 2026-04-27

**Authors:** L. H. HUSSEIN, S. A. AL-MUFFTI

**Affiliations:** Department of Biology, College of Science, University of Duhok, Duhok, 42001 Kurdistan Region, Iraq; Department of Biology, College of Science, University of Duhok, Duhok, 42001 Kurdistan Region, Iraq

**Keywords:** *E. granulosus*, molecular detection, phylogenetic tree analysis

## Abstract

Cystic hydatid disease, caused by *Echinococcus granulosus*, is a major zoonotic infection with significant public health and economic impact in endemic regions, including Iraq. This study aimed to detect the mitochondrial cytochrome c oxidase subunit I (COI) gene in *E. granulosus* and to characterise hydatid cysts based on morphological and pathological features, including cyst wall layers, cyst type, and organ involvement, in Duhok Governorate, the Kurdistan Region of Iraq (KRI). In this study, characterisation refers to the classification of hydatid cysts by type (fertile, infertile, or calcified) and organ involvement. Of the 58 hydatid cyst samples collected, 17 were from humans, and 41 were from sheep. Cysts were classified according to organ involvement and cyst type (fertile, infertile, or calcified). Among sheep samples, 75.6 % were fertile, 21.9 % infertile, and 2.4 % calcified, whereas human samples showed 82.4 % fertile, 5.9 % infertile, and 11.8 % calcified cysts. Molecular detection using PCR targeting the COI gene confirmed the presence of *E. granulosus* DNA in 40 sheep and 15 human samples. Nineteen PCR-positive samples were selected for sequencing, resulting in 13 high-quality sequences (three human and ten sheep isolates) that were successfully submitted to GenBank. Statistical analysis showed no significant association between host type and host gender, infection type, or PCR positivity (p > 0.05). However, significant associations were observed between cyst type and organ involvement, cyst type and PCR results, and PCR results and affected organs (p < 0.05). Overall, the findings support the usefulness of COI-based PCR for molecular detection of *E. granulosus* and highlight the value of integrating cyst characterisation with molecular approaches to improve epidemiological understanding and support control strategies in endemic areas.

## Introduction

Cystic echinococcosis (CE), caused by the larval stage of *Echinococcus granulosus*, is an endemic zoonotic infection in Iraq, particularly in the Kurdistan Region. The disease represents a major public health and economic concern due to its significant impact on human health and livestock production, mainly sheep, which serve as the principal intermediate hosts in the region (Alhadidi, *et al*., 2022).

In the Duhok Governorate, close interactions among humans, livestock, and domestic dogs, together with traditional backyard slaughtering practices, facilitate the continuous transmission of CE. Several hospital-based and abattoir studies conducted in northern Iraq have reported hydatid cyst infections in humans and sheep, with the liver being the most frequently affected organ and fertile cysts commonly observed in sheep hosts (Habib & Abdullah, 2025; Issa *et al*,. 2022). These results highlight the persistent transmission of *E. granulosus i*n this area and suggest the necessity for more extensive epidemiological studies. followed by pulmonary and other organ infections, demonstrating the consistent organ-specific predilection of EC across different regions (Nasralla *et al*., 2025).

The integration of molecular detection with the characterisation of cyst fertility and organ tropism provides a comprehensive framework for assessing parasite viability and transmission potential. Recent epidemiological studies in Iraq have emphasised the importance of combining molecular approaches with cyst characterisation to improve understanding of local CE transmission dynamics (Babawat, 2024).

It is essential to accurately detect *E. granulosus* to understand the epidemiology and transmission dynamics of CE in endemic regions. Conventional diagnostic approaches that depend on imaging techniques and macroscopic examination of cysts may prove insufficient for confirming parasite identity. Molecular detection techniques, particularly polymerase chain reaction (PCR), targeting mitochondrial genes such as cytochrome c oxidase subunit I (COI), have been widely used as reliable tools for detecting *E. granulosus* (Bowles *et al*,. 1992; Thompson, 2017).

However, despite the endemic nature of CE in Duhok Governorate, data on molecular detection and detailed characterisation of hydatid cysts in humans and sheep remain limited. Therefore, this study aimed to detect the COI gene *of Echinococcus granulosus* in human and sheep hydatid cysts from the Duhok, Kurdistan Region, Iraq, using PCR.

## Material and Methods

### Sample Collection

**Fig. 1.**
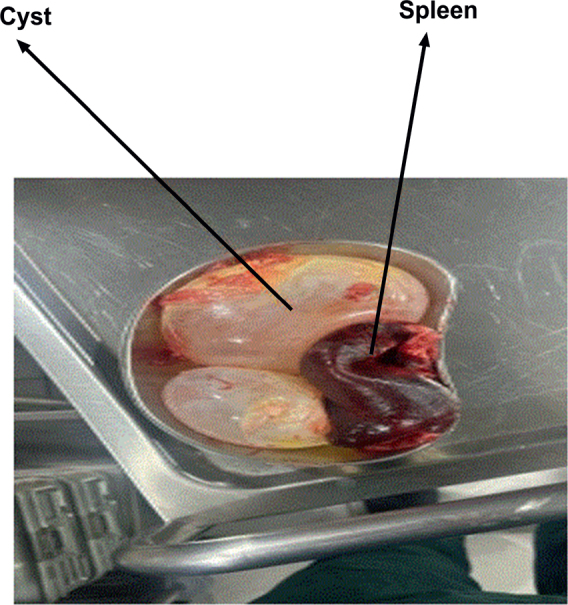
The macroscopic characteristics of hydatid cysts in the human spleen highlight cyst morphology and organ involvement associated with cystic echinococcosis in humans.

**Fig. 2.**
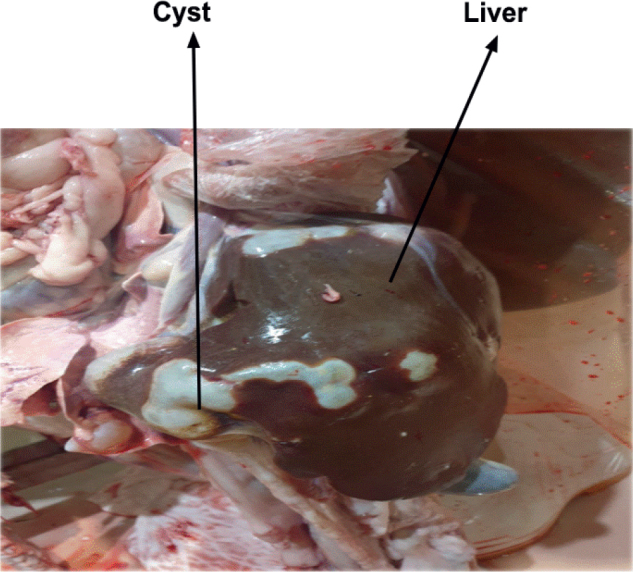
Hydatid cysts in a sheep’s liver are classified into different cyst types and evaluated for their fertility.

Samples of hydatid cysts were taken from sheep and human patients in the Kurdistan Region of Iraq’s Duhok Governorate between November 2024 and February 2025. Human cyst samples were obtained from male and female patients undergoing surgical procedures at Azadi Teaching Hospital, Vin Hospital, Duhok Private Hospital, and Shiryan Hospital. These hospitals serve as major referral centres for cystic echinococcosis in Duhok City and its neighbouring districts, including Zakho, and accept patients from both urban and rural areas. Human cysts were surgically removed from affected organs, primarily the liver and spleen, as shown in Figure 1. Hydatid cyst samples were collected from sheep slaughtered at the Duhok and Zakho abattoirs. The cysts were isolated from various organs, including the liver, lungs, and kidneys, in both male and female sheep, as shown in Figure 2. These abattoirs were chosen because of their significant role in routine meat inspection and their convenient access to livestock from various regions within the governorate. All collected cyst samples were placed in sterile containers filled with normal saline and transported to the Parasitology Laboratory under aseptic conditions for further examination.

### Morphological Examination of Hydatid Cysts

**Fig. 3.**
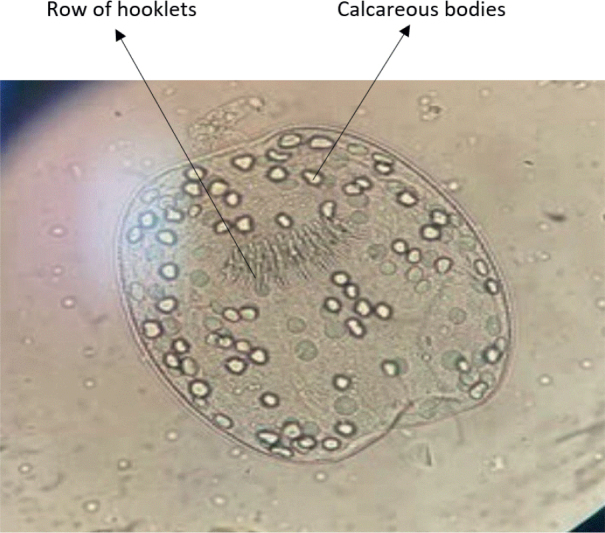
A light microscopic image (×40 magnification) illustrating protoscolices obtained from a viable hydatid cyst in sheep liver, thereby affirming cyst viability and corroborating fertility classification.

Hydatid cysts were examined by direct observation and by light microscopy. The cysts were grouped as fertile, infertile, or calcified according to their developmental stage. Cyst viability was assessed by aseptically collecting cyst fluid and centrifuging it at 3000 rpm for 10 minutes, as outlined by Barazesh *et al*. (2020). Protoscolices were identified in the sediment post-centrifugation via light microscopy examination. Cysts containing protoscolices were considered fertile, as illustrated in Figure 3. Protoscolices were separated for further analysis, while germinal layers were taken from infertile cysts. Calcified cysts were identified by their firm texture and the absence of viable contents.

### DNA Extraction Method

Genomic DNA was independently extracted from hydatid cyst specimens, including protoscolices from fertile cysts and germinal layers from infertile cysts. Before extraction, samples were meticulously rinsed with sterile phosphate-buffered saline (PBS) to eliminate any remaining cyst fluid. DNA extraction was performed using the AddPrep Genomic DNA Extraction Kit (Addbio, Korea) according to the manufacturer’s instructions. About 20 mg of germinal layer tissue or 100 – 200 μl of protoscolices suspension was placed into a 1.5 ml microcentrifuge tube, combined with lysis buffer and proteinase K, and incubated at 56°C until complete tissue lysis occurred. The DNA concentration and purity were assessed using a NanoDrop spectrophotometer (Thermo Scientific, USA) by measuring absorbance at 260 nm and 280 nm. DNA samples were stored at -20°C until further molecular analysis.

### PCR Amplification of the COI Gene and Gel Electrophoresis Method

A polymerase chain reaction (PCR) utilising species-specific primers (SH/LU) was performed to amplify a segment of the mitochondrial cytochrome c oxidase subunit I (COI) gene of *E. granulosus*. The COI gene, present in multiple copies in mitochondrial DNA, was chosen for species-level molecular detection of *E. granulosus* (Bowles *et al*,. 1992; Nakao *et al*,. 2007).

A 25 μl reaction volume was employed for each sample, comprising 2 μl of genomic DNA. A 213 bp fragment of the COI gene was amplified using this primer set. 1 μl of forward primer SH (5’-GCAGGGTTTGGGGTCATCAT-3’), 1 μl of reverse primer LU (5’-CCGTAACTCCCCCAAACGTA-3’), and Table 1 showing species-specific primers of the Mt-COI gene for *E. granulosus*. 12.5 μl of PCR master mix, and 8.5 μl of nuclease-free water (sterile injection water).

**Table 1.** The designed species-specific primers of the Mt-COI gene for *E. granulosus*

Primer	Sequence (5'->3')	No. of nucleotides	Size gene product
**Mt-COI SH**	GCAGGGTTTGGGGTCATCAT	20	213bp
**Mt-COI LU**	CCGTAACTCCCCCAAACGTA		

The PCR amplification process began with an initial denaturation at 95°C for 10 minutes, followed by 35 cycles comprising denaturation at 95°C for 45 seconds, annealing at 55°C for 45 seconds, and extension at 72°C for 1 minute. The process concluded with a final extension at 72°C for 5 minutes.

**Fig. 4.**
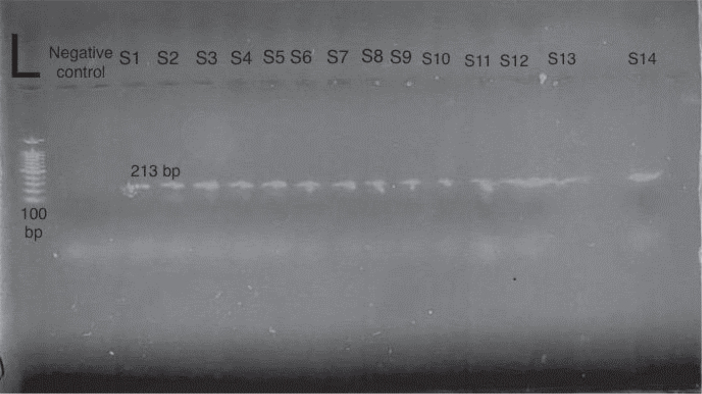
An agarose gel electrophoresis image showing PCR amplification of the mitochondrial cytochrome c oxidase subunit I (COI) gene of *Echinococcus granulosus* from human hydatid cyst samples, visualised under ultraviolet (UV) illumination. Lane L represents the 100 bp DNA molecular weight ladder. Lane negative control. Lanes S1–S14 correspond to human samples, with positive samples showing a distinct band at the expected size of 213 bp.

**Fig. 5.**
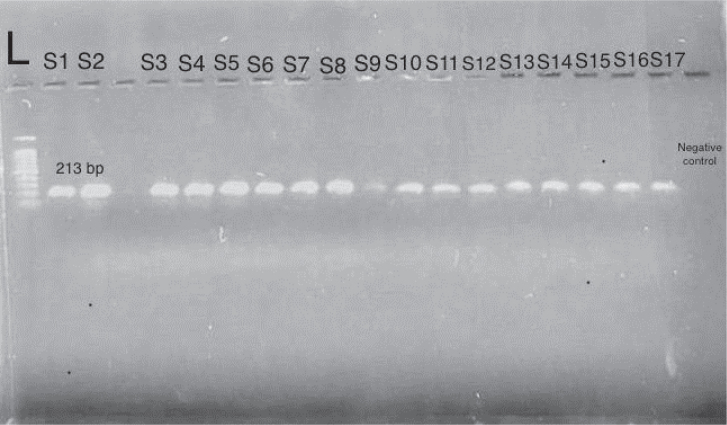
An agarose gel electrophoresis image showing PCR amplification of the mitochondrial cytochrome c oxidase subunit I (COI) gene of *Echinococcus granulosus* from sheep hydatid cyst samples, visualised under ultraviolet (UV) illumination. Lane L represents the 100 bp DNA molecular weight ladder. Lanes S1–S17 correspond to sheep samples, with positive samples showing a distinct band at the expected size of 213 bp. The negative control shows no amplification.

PCR products were analysed by agarose gel electrophoresis. The gel was prepared by dissolving 0.5 g of agarose in 40 ml of distilled water and 10 ml of 1× TBE buffer, then heating until fully dissolved. After cooling, 5 μl of safe DNA dye was added. Electrophoresis was carried out at 45 V for 15 minutes and then at 85 V for 30 minutes. DNA bands were observed under ultraviolet light, and fragment sizes were approximated using a 100 bp DNA ladder, as depicted in Figure 4 for humans and Figure 5 for sheep.

### DNA Sequencing Analysis Method

DNA from samples that tested positive for *E. granulosus* was selected for sequencing following PCR confirmation of the amplified COI gene fragment. In total, nineteen PCR-positive samples were subjected to DNA sequencing. Purified PCR amplicons were sequenced using Sanger dideoxy sequencing technology at a specialised molecular facility in the Republic of Korea.

The obtained sequences were carefully evaluated for quality and accuracy. Thirteen high-quality sequences, including three human and ten sheep isolates submitted to GenBank, were used for sequence comparison and the construction of a phylogenetic tree to assess the accuracy of COI-based molecular detection by comparing with reference sequences.

### Phylogenetic Analysis Method

Two separate phylogenetic analyses were performed based on partial COI gene sequences: one for human isolates and one for sheep isolates.

**Fig. 6.**
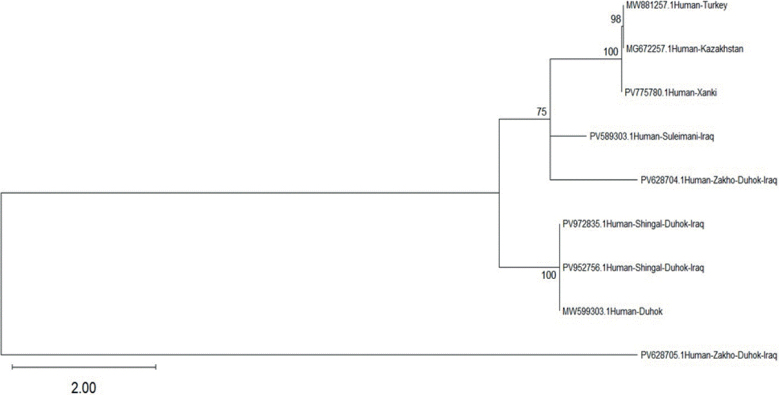
Phylogenetic tree based on partial mitochondrial COI gene sequences showing the genetic relationships among *Echinococcus granulosus* isolates obtained from human samples and reference sequences from different geographic regions. The tree was constructed using the Maximum Likelihood method.

**Fig. 7.**
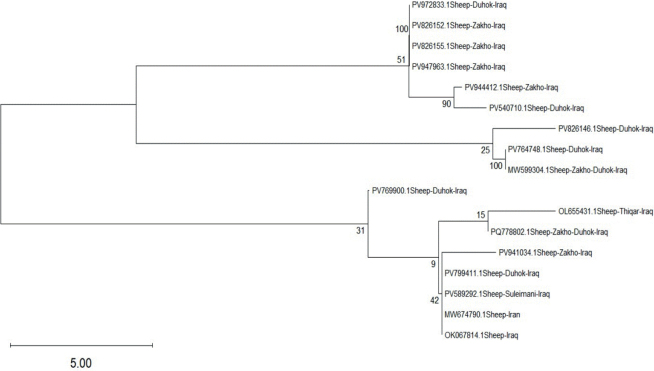
Phylogenetic tree based on partial mitochondrial COI gene sequences showing the genetic relationships among *Echinococcus granulosus* isolates obtained from sheep samples and reference sequences from different geographic regions. The tree was constructed using the Maximum Likelihood method.

Figures 6 and 7, showing the phylogenetic analyses for humans and sheep, were conducted using the Maximum Likelihood (ML) method under the Tamura–Nei model (Tamura & Nei, 1993). The robustness of the tree topology was assessed by bootstrap analysis with 1,000 replicates (Felsenstein, 1985). Trees were initially generated automatically utilising the Neighbour-Joining (NJ) and BioNJ algorithms (Saitou & Nei, 1987), based on pairwise distance matrices estimated according to the Tamura–Nei model.

The phylogenetic analysis of human isolates included three partial COI gene sequences from human samples, together with six reference sequences. In contrast, the phylogenetic analysis of sheep isolates included 10 partial COI sequences from sheep samples and 7 reference sequences. In both analyses, all positions with gaps or missing data were removed using the complete deletion option, resulting in a final dataset of 161 nucleotide positions. Phylogenetic analyses were conducted using MEGA version 12 (Kumar *et al*,. 2024).

## Ethical Approval and/or Informed Consent

Ethical approval for this study was obtained from the Veterinary Directorate in Duhok, Kurdistan Region, Iraq, to collect sheep samples. Human hydatid cyst samples were approved for collection by the General Directorate of Health in Duhok (Approval No. 3010-2024-9-54, dated October 30, 2024).

All procedures involving humans adhered to institutional and internationally recognised ethical guidelines, including the principles outlined in the Helsinki Declaration. Sample collection was performed anonymously, and no identifying personal information was disclosed.

Written informed consent was obtained from all participants or their legal guardians prior to sample collection. Strict confidentiality was maintained throughout the study, and demographic and clinical data, such as age, gender, infection site, and residence, were collected solely for research purposes.

## Result

### Morphological Characteristics of Hydatid Cysts

A total of 58 hydatid cysts were obtained from sheep (n = 41) and humans (n = 17). The cysts were sorted into groups based on the organ affected and cyst type (fertile, infertile, or calcified). The primary classification of sheep cysts was fertile (75.6 %), followed by infertile (21.9 %) and calcified (2.4 %). Fertile cysts constituted 82.4 % of human samples, calcified cysts made up 11.8 %, and infertile cysts accounted for 5.9 %. The liver was the most affected organ in both hosts, followed by the spleen in humans and the lungs and kidneys in sheep. Table 2: Classified hydatid cyst samples were collected according to host species, number from infected organs, and cyst types.

**Table 2.** Distribution of hydatid cysts by host, organ, and cyst type, showing predominance of liver involvement and fertile cysts in both sheep and human samples.

Host	Sex	Organ	Fertile	Infertile	Calcified	Total
**Animal**						
Sheep	Female	Liver	13	6	-	19
Sheep	Male	Liver	11	2	-	13
Sheep	Female	Lung	4	1	-	5
Sheep	Male	Lung	3	-	-	3
Sheep	Female	Kidney	-	-	1	1
**Human**						
Human	Female	Liver	7	-	1	8
Human	Male	Liver	6	1	1	8
Human	Male	Spleen	1	-	-	1

Macroscopic examination enabled the identification of the unique cyst wall and the fluid within hydatid cysts. Microscopic examination confirmed the presence of protoscolices in fertile cysts, while infertile cysts lacked these structures. Moreover, the calcified cysts showed stiff structures devoid of any functional elements.

*Molecular Detection Results of Echinococcus granulosus by PCR* PCR analysis of the COI gene successfully detected *E. granulosus* DNA in 55 of 58 samples. Fifteen out of seventeen human samples demonstrated positive amplification, while forty out of forty-one cysts in sheep samples tested positive through PCR.

### Results of DNA Sequencing and Phylogenetic Analysis

Of the PCR-positive samples, 19 isolates were selected for DNA sequencing. Quality assessment yielded 13 high-quality partial COI gene sequences, including three human isolates and ten sheep isolates, which were used for comparative sequence analysis and phylogenetic reconstruction. The obtained sequences were deposited in the GenBank database under accession numbers PV775780, PV952756, and PV972835 (human isolates) and PV972833, PV826152, PV826155, PV947963, PV944412, PV764748, PV941034, PV769900, PV826146, and PV799411 (sheep isolates). All illustrated in Table 3, accession no. of human, and Table 4, accession no. of sheep, with accession numbers from another region inside the Kurdistan region, Iraq, and neighboring countries.

**Table 3.** Illustrated Accession number recorded by NCBI GenBank in human and explain identified percentage with another accession number, (the accessions obtained in the present study and submitted to the Gene Bank are indicated in bold).

Species	Accession No.	Region	Explain Identified %
***E. granulosus***	**PV775780**	**Khanke**	Only PV775780 appeared similarity in NCBI with another accession no. 97%. But PV952756 & PV972835 appeared similarity in NCBI with another by 98-100%.
***E. granulosus***	**PV952756**	**Sinjar**	
***E. granulosus***	**PV972835**	**Sinjar**	
*E. granulosus*	MW881257	Turkey	
*E. granulosus*	MG672257	Kazakhstan	
*E. granulosus*	PV589303	Sulaymaniyah	
*E. granulosus*	PV628705	Zakho	
*E. granulosus*	MW599303	Duhok	
*E. granulosus*	PV628704	Zakho	

**Table 4.** Illustrated Accession number recorded by NCBI GenBank in sheep and explain the identified percentage with another accession number (the accessions obtained in the present study and submitted to the Gene Bank are indicated in bold).

Species	Accession no.	Isolated region	Similarity%
***E. granulosus***	**PV764748**	**Duhok**	------------
***E. granulosus***	**PV769900**	**Duhok**	------------
***E. granulosus***	**PV826152**	**Zakho**	------------
***E. granulosus***	**PV826155**	**Zakho**	------------
***E. granulosus***	**PV799411**	**Duhok**	------------
***E. granulosus***	**PV826146**	**Duhok**	------------
***E. granulosus***	**PV941034**	**Zakho**	------------
***E. granulosus***	**PV944412**	**Zakho**	------------
***E. granulosus***	**PV947963**	**Zakho**	------------
***E. granulosus***	**PV972833**	**Duhok**	------------
*E. granulosus*	PV540710	Duhok	100%
*E. granulosus*	OL655431	Thi-qar	100%
*E. granulosus*	OK067814	Iraq	100%
*E. granulosus*	PV589292	Sulaymaniyah	100%
*E. granulosus*	PQ778802	Zakho	100%
*E. granulosus*	MW599304	Zakho	98%
*E. granulosus*	MW674790	Iran	98%

We generated separate phylogenetic trees for human and sheep isolates based on partial COI sequences. In both analyses, the sequences produced in this study exhibited a close clustering with reference *E. granulosus* sequences from various geographic regions. This clustering pattern demonstrates that the correlation of current isolates with previously documented sequences enhances the reliability of COI-based molecular detection in both human and sheep samples.

### Statistical Analysis Results

Statistical analyses were conducted using SPSS version 25. Associations between categorical variables were evaluated using p-values; p < 0.05 was regarded as statistically significant, and values ≥ 0.05 were considered non-significant. No statistically significant associations were observed between host type (human vs. sheep) and host gender (p = 0.33), infection type (p = 0.17), or PCR results (p = 0.144). In contrast, a statistically significant association was identified between cyst type and the affected organ (p = 0.004) and between cyst type and PCR results (p < 0.001). Additionally, PCR results were significantly associated with the affected organ (p < 0.001). Additionally, a significant correlation between organ involvement and host type was found (p = 0.03), suggesting that infection patterns differed between sheep and human hosts.

## Discussion

This study offers a comprehensive evaluation of cystic echinococcosis in the Duhok Governorate by combining morphological observations, molecular detection, statistical analysis, and phylogenetic analysis of *E. granulosus* isolates from humans and sheep. This combined approach enhances understanding of the local epidemiological situation and enables meaningful comparison with findings reported from other endemic regions.

Morphological examination demonstrated that the liver was the most frequently affected organ in both humans (94.12%) and sheep (78.05 %). These proportions are higher than those reported in central and southern Iraq, where hepatic involvement in sheep generally ranged from 55 % to 70 % (Jasim *et al*,. 2024). Comparable patterns have been observed in western Iran, where liver cysts accounted for over 80 % of human cases (Moosazadeh *et al*,. 2017). The prevalence of hepatic infection corresponds biologically with the life cycle of *E. granulosus*, as the liver functions as the principal filtration barrier for oncospheres entering the portal circulation. A statistically significant association between host type and organ involvement (p = 0.03) indicates disparities in infection patterns between humans and sheep, likely reflecting differences in exposure pathways, immune responses, and disease progression. Furthermore, a high proportion of fertile cysts was recorded in both sheep (75.6 %) and humans (82.4 %). The observed fertility rates are higher than those reported in other endemic regions, including Iraq and Iran, where cyst fertility in sheep shows considerable variation across studies (Farhood *et al*,. 2022; Shahnazi *et al*,. 2013). The increased fertility observed in sheep supports their role as the primary intermediate host and a significant factor in parasite transmission. The protracted asymptomatic development of cysts and unintentional consumption of parasite eggs in humans may explain the existence of viable cysts due to delayed diagnosis. The significant correlation between cyst fertility and PCR positivity is likely due to fertile cysts producing greater amounts of parasite DNA. Molecular detection via PCR targeting a 213-bp fragment of the mitochondrial COI gene showed high detection rates in both human and sheep isolates. These findings are consistent with previous studies from Iran and Turkey that utilized mitochondrial markers for the detection of *Echinococcus granulosus* (Barazesh *et al*,. 2020). The findings validate the COI gene’s effectiveness as a reliable molecular marker for detecting *E. granulosus* in epidemiological studies. The significant correlations observed among cyst type, organ involvement, and PCR results highlight the inherent relationship between cyst biological characteristics and their molecular detectability.

All isolates were classified within the *E. granulosus* lineage based on their partial mitochondrial COI gene phylogeny, with human isolates PV775780, PV952756, and PV972835 being closely related to those from Iraq and neighbouring countries. PV775780, sourced from Khanke–Duhok, is phylogenetically aligned with reference isolates from Turkey (MW881257) and Kazakhstan (MG672257), demonstrating robust bootstrap support (98 – 100 %), indicating significant genetic affinity across disparate geographical regions and suggesting either a shared ancestral lineage or the continuous spread of genetically stable parasite populations, while this have 97 % blast identify value confirms the strong genetic link, even though the few nucleotide alterations discovered may be the consequence of host-induced or environmental adaptation mechanisms (Romig *et al*,. 2017).

The two remaining human isolates (PV952756 and PV972835) formed a distinct and robust sub-cluster with a previously documented local isolate (MW599303), exhibiting 100 % bootstrap support. This robust clustering strongly suggests the existence of a stable, localised transmission cycle for *E. granulosus* in the Duhok Governorate (Issa *et al*., 2022).

The phylogenetic analysis of sheep isolates revealed close genetic relationships with regional reference sequences. These findings confirm the existence of a genetically conserved parasite population among livestock in the Kurdistan Region, as the ten sheep isolates submitted to GenBank (PV972833, PV826152, PV826155, PV947963, PV944412, PV764748, PV941034, PV769900, PV826146, and PV799411) cluster predominantly with isolates from Iraq and neighbouring countries. The presence of a dominant lineage among sheep hosts was further supported by a well-defined clade comprising PV972833, PV826152, PV826155, and PV947963, showing maximal bootstrap support (100).

Clustering between PV944412 and the reference isolate PV540710 was supported by a bootstrap value of 90, whereas PV764748 grouped with MW599304 with maximal bootstrap support (100), indicating substantial genetic similarity. In contrast, the sheep isolate PV769900 formed a separate branch within this parasite and showed lower bootstrap values (15 – 42) than those of reference isolates from Thi-Qar, Iraq (OL655431), and Iran (MW674790). This reduced support is more consistent with limited nucleotide variation than with true phylogenetic divergence. It may reflect the influence of animal movement, geographic isolation, host adaptation, or localised environmental pressures.

Bootstrap analysis played a key role in evaluating the robustness of the phylogenetic tree. Elevated bootstrap values (≥70 %) demonstrate strong statistical support for the inferred branching patterns, whereas values close to 100 % provide compelling evidence for common evolutionary ancestry. Conversely, reduced bootstrap values indicate lower confidence in specific nodes. They are more likely to be explained by limited sequence length or low levels of genetic variation rather than genuine phylogenetic divergence. Taken together, the strong bootstrap-supported clustering and short genetic distances indicate low to moderate genetic variability, reflecting a stable transmission cycle of this infection in northern Iraq.

The close genetic relationship between humans and sheep isolates highlights the important epidemiological link between livestock and human infections, demonstrating the crucial role of sheep in sustaining transmission cycles in the Duhok Governorate.

A limitation of this study is the relatively short time it took to collect data. This study focused on identifying a mitochondrial COI gene fragment to classify *Echinococcus granulosus* at the species level. Therefore, a complete analysis of genotypes was not included. Future studies should include several mitochondrial gene targets. This will improve how we identify genotypes and enable a more thorough assessment of genetic diversity within *Echinococcus granulosus*. Therefore, the current findings provide important basic information for identifying the molecular aspects and studying the spread of cystic echinococcosis in the Duhok Governorate.

## Conclusion

This study provides a comprehensive assessment of *Echinococcus granulosus* in Duhok Governorate by integrating morphological examination, molecular detection, statistical analysis, and phylogenetic analysis of isolates derived from humans and sheep. The elevated fertility rates of hydatid cysts in sheep (75.6 %) and humans (82.4 %), coupled with the prevalence of hepatic involvement, underscore the significance of sheep as a vital intermediate host and suggest ongoing transmission in the area.

*E. granulosus* DNA was detected in the majority of samples through molecular analysis using COI-based PCR. Phylogenetic studies have shown that *E. granulosus* sequences from Iraq and nearby areas are closely related to those from sheep and humans. The evidence suggests a genetically similar group of parasites in the area. However, the short gene fragment analysed was sufficient for species-level molecular detection but limits definitive genotype or strain-level identification.

Overall, the findings indicate sustained transmission of cystic echinococcosis in Duhok Governorate and drive home the importance of integrated control strategies, including improved abattoir hygiene, management of stray dogs, public health education, and continued molecular surveillance. Future studies employing longer mitochondrial regions or multilocus approaches are recommended to enable more detailed genetic characterisation and support more effective control programs.
